# Facial Motion Enhances Face Recognition and Guides Visual Attention in Typical Recognisers and Developmental Prosopagnosia

**DOI:** 10.3390/brainsci16060567

**Published:** 2026-05-27

**Authors:** Laura Sexton, Natalie Butcher, Jonathon Reay

**Affiliations:** 1 School of Psychology, University of Sunderland, Sunderland SR1 3SD, UK; 2 School of Social Sciences, Humanities and Law, Teesside University, Middlesbrough TS1 3BX, UK; j.reay@tees.ac.uk

**Keywords:** developmental prosopagnosia, face recognition, motion, eye-movements

## Abstract

**Highlights:**

**What are the main findings?**

- Facial motion improves face recognition accuracy in both individuals with developmental prosopagnosia (DP) and neurotypical controls.
- Motion increases attention to internal facial features (eyes, nose, mouth) in both individuals with and without DP; however, individuals with DP differ from controls in how attention is distributed across these features.

**What are the implications of the main findings?**

- Facial motion promotes more effective face-scanning strategies by directing attention to diagnostic internal features.
- Dynamic faces may have value in assessment or remediation approaches aimed at supporting face recognition in individuals with DP.

**Abstract:**

**Background:** Facial motion has been shown to enhance face recognition in typical recognisers and increase attention to diagnostic internal facial features (eyes, nose and mouth), which may support improved recognition. The present study aimed to replicate these effects and assess whether they extend to individuals with developmental prosopagnosia (DP), who show impairments in face recognition and reduced attention to the internal features. **Methods:** Participants completed a famous face recognition task (Experiment 1) and an unfamiliar old/new recognition task (Experiment 2), with both static and moving stimuli. Eye movements were recorded to assess visual attention. In each experiment, two analyses were conducted: a replication analysis in a neurotypical sample (Experiment 1: *n* = 49; Experiment 2: *n* = 51), and a separate comparison of 14 individuals with DP with a subset of 16 age-matched controls. **Results:** Across both experiments, recognition accuracy was higher for moving than static faces in both the control and DP samples, and participants directed a greater proportion of visual attention to internal facial features when faces were presented in motion. However, compared to age-matched controls, individuals with DP showed differences in the allocation of attention to the internal facial features. **Conclusions:** The findings replicate evidence that facial motion enhances recognition and increases attention to the internal facial features in typical recognisers and extend these effects to individuals with DP. Although individuals with DP benefit from motion, differences in the distribution of attention across internal features remain, suggesting that motion alters but does not normalise face-scanning strategies.

## 1. Introduction

### 1.1. The Motion Advantage in Face Recognition

Faces are a uniquely important category of visual stimuli, and the ability to perceive and recognise them is among the most highly developed perceptual skills that humans possess [1]. Face recognition plays a critical role in both applied and everyday contexts. In applied settings, failures of recognition can have serious consequences, such as allowing a criminal to evade identification [2] or permitting the use of fraudulent identification documents [3]. In everyday life, accurate face recognition supports effective social interaction by enabling rapid access to information about a person’s identity, emotional state, and intentions, which in turn guides appropriate behavioural responses [4].

One factor that can enhance face recognition is facial motion. A face can exhibit rigid motion, in which the head changes orientation while maintaining its structure (e.g., nodding or shaking the head) and non-rigid motion, in which individual facial features move relative to one another (e.g., speech or emotional expressions) [5]. Seeing a face in motion improves unfamiliar face learning [6,7,8], face matching [9], and familiar face recognition [10,11]. This effect is commonly referred to as the motion advantage (see Xiao et al. for a review [12]).

Several theoretical accounts have been proposed to explain the motion advantage. First, the supplemental information hypothesis (SIH [13]) proposes that idiosyncratic patterns of facial motion, known as dynamic identity signatures, provide additional cues that support recognition. According to this view, familiar faces can be recognised not only from their static properties, such as facial features and overall structure, but also from the distinctive ways in which they move. These characteristic movements are thought to vary between individuals in their spatiotemporal properties, such that the particular way a person smiles, speaks, or moves their head may itself become a cue to identity [14].

Evidence for the SIH comes from studies using facial stimuli in which motion cues are separated from static structural information. Hill and Johnston [15] superimposed learned patterns of facial motion onto average head models and found that participants could accurately extract identity information from rigid head movement alone. However, the motion advantage for familiar faces is most commonly observed under non-optimal viewing conditions, such as blur, negation, or reduced image quality [16,17,18], suggesting that dynamic identity signatures function as a supplementary cue to familiar face recognition when static information is degraded or unreliable.

A second theory, the representation enhancement hypothesis (REH [13]), focuses on the role of motion in the learning of unfamiliar faces. According to this view, facial motion facilitates recognition by supporting the formation of a more robust structural representation of a face than would be obtained from a single static image. This benefit appears to extend beyond simply viewing multiple snapshots of the same face: recognition is better following exposure to a rigidly moving face than to a series of static images extracted from the same video sequence [8]. Learning from non-rigid motion has also been shown to support recognition across changes in viewpoint [19], consistent with the idea that the resulting representation is more structurally robust and less tied to a specific image. Importantly, some studies suggest that the benefits of motion are strongest at encoding rather than at test, which fits closely with the central claim of the REH that motion assists in building the initial representation of an unfamiliar face [6].

Finally, the social signals hypothesis (SSH [20]) emphasises the role of attention in explaining the motion advantage. This account proposes that socially meaningful information embedded in non-rigid facial movement—such as eye gaze, emotional expressions, and speech-related actions—influences how attention is allocated during face processing. In principle, this information may either facilitate or interfere with identity recognition. On the one hand, socially relevant motion may guide attention towards identity-diagnostic regions of the face. On the other hand, processing these social cues may impose additional cognitive demands that compete with identity processing. As such, the effects predicted by this account are likely to be context dependent.

Although Roark et al. [20] did not specify which facial regions are most likely to receive increased attention, subsequent interpretations have suggested that non-rigid facial motion may preferentially direct attention towards the internal facial features, namely the eyes, nose, and mouth [10,21]. This is important because the internal features are both highly informative for identity recognition and the primary source of socially meaningful facial movement. While the SSH provides a plausible attentional account of the motion advantage, direct empirical support for its underlying assumptions remains limited, making them an important target for empirical investigation in the current series of experiments.

### 1.2. Visual Attention and Face Processing

Eye movements provide a useful index of how attention is allocated during face processing [22]. A large body of research has demonstrated that the internal facial features are critical for accurate identity recognition [23,24], and greater attention to these regions is associated with improved performance [25,26]. Facial motion may influence this distribution of attention, as motion is known to capture attention [27], and non-rigid facial movements are concentrated within the internal features and convey socially relevant information [28,29]. Taken together, these findings suggest that motion may enhance recognition by directing attention towards diagnostic regions of the face.

Recent work has begun to examine this possibility more directly. In our previous work [30], we recorded eye movements while participants completed familiar and unfamiliar face recognition tasks using both static and predominantly non-rigid moving stimuli. Across multiple experiments, participants allocated a greater proportion of fixations to the internal facial features when faces were presented in motion. Attention to internal non-feature regions (e.g., chin, cheeks, forehead) and external regions (e.g., hair and ears) was correspondingly reduced. Notably, during familiar face recognition, the magnitude of the motion advantage was positively associated with the proportion of fixations directed to the internal features, suggesting a functional link between attentional allocation and recognition performance. These findings provide evidence that non-rigid facial motion may enhance recognition by directing attention towards the identity-diagnostic regions of the face.

### 1.3. Replicating and Extending Previous Findings

The first aim of the present study was to replicate these findings in a sample of typical recognisers, during the recognition of familiar (Experiment 1) and learning of unfamiliar (Experiment 2) faces. Establishing the robustness of these effects is important before extending this framework to populations characterised by atypical face-processing strategies (e.g., individuals with developmental prosopagnosia). As our previous research found little difference between internal non-feature and external regions, here we collapsed these regions into a single interest area, termed ‘external features’. Internal features were defined as the eyes, nose, and mouth.

Although our previous findings demonstrate that non-rigid motion increases the proportion of time and fixations directed towards the internal features, they do not unambiguously indicate how attention is allocated across the face. On the one hand, the salient movement of the eyes, nose, and mouth may attract and maintain attention to the internal features, consistent with the assumption that motion biases attention towards identity-relevant regions. On the other hand, when viewing a moving face, participants may engage in more extensive sampling across the face as a whole. In this case, the increased attention directed to the internal facial features may result from their central location rather than reflecting sustained attention to these regions. This latter interpretation is consistent with eye-tracking findings reported by Xiao et al. [31], who observed that non-rigid motion increased both the total number of fixations to a face and the number of fixation shifts between salient internal features in infants, although this may not generalise to older populations. The present study, therefore, sought to distinguish between these possibilities by examining the number of attentional shifts between facial features. If motion serves to maintain attention, it should result in fewer fixation shifts across the face.

Based on our previous findings, we predicted that participants would demonstrate improved recognition performance for moving faces compared to static faces. In addition, motion was expected to increase the proportion of time and fixations directed towards the internal features relative to the external features. Finally, if motion maintains attention on the internal features, moving faces should be associated with fewer shifts between key facial features.

### 1.4. Extending the Motion Advantage to Developmental Prosopagnosia

While the findings of Butcher et al. [30] provide evidence that non-rigid facial motion may enhance recognition by directing attention towards diagnostic regions of the face, an important question is whether this mechanism extends to individuals with developmental prosopagnosia (DP). DP is characterised by a selective impairment in face recognition in the absence of neurological damage or broader cognitive deficits [32,33]. Individuals with DP frequently report difficulties recognising familiar people in everyday life and often rely on alternative cues, such as voice, clothing, or gait, to identify others [34].

A key characteristic of DP, particularly relevant to the current study, is the atypical allocation of visual attention during face processing. Compared to typical observers, individuals with DP direct less attention to the internal facial features and more attention to external features such as hair and face shape [25,35]. Given that internal features are most diagnostic for identity, this pattern of attention may contribute to their recognition impairments. Importantly, this atypical scanning behaviour appears to be modifiable. Training studies have demonstrated that directing attention towards internal features can improve recognition performance and, in some cases, lead to more typical patterns of face-scanning [36,37]. These findings suggest that interventions which influence visual attention may provide a route to improving face recognition in DP.

Facial motion may offer one such mechanism. As outlined above, non-rigid motion captures attention and biases it towards the internal facial features in typical observers [30]. If similar effects occur in individuals with DP, motion may serve to ‘normalise’ face-scanning behaviour by increasing attention to identity-diagnostic regions of the face. However, the extent to which individuals with DP can utilise motion-based information remains unclear.

There is some evidence that aspects of motion processing are relatively typical in prosopagnosia. Individuals with DP perform typically on tasks involving biological motion, including expression recognition and lip-reading [38,39], suggesting that neural systems involved in processing dynamic facial information may function typically. This raises the possibility that motion could support face recognition even when static processing is impaired.

Despite this, few studies have examined the role of motion in face recognition in DP, and findings to date are mixed. Some suggest that individuals with DP can benefit from motion when learning or matching unfamiliar faces, particularly when motion provides rigid structural information [5,40]. However, this benefit is not consistently observed across individuals [41,42]. Importantly, most of this work has focused on unfamiliar faces, leaving open the question of whether motion also facilitates the recognition of familiar identities. To date, only one study has directly examined the effect of motion on familiar face recognition in DP. Bennetts et al. [10] found that, at a group level, individuals with DP showed improved recognition of famous faces when presented in motion compared to static. While these findings suggest that motion can facilitate recognition in DP, the mechanisms underlying this effect remain unclear.

Critically, no previous research has examined whether any motion advantage observed in DP can be explained by changes in visual attention. Given the aforementioned findings, it is possible that non-rigid motion enhances recognition in DP by altering face-scanning strategies, a hypothesis that is yet to be tested. The second aim of the present study was therefore to examine whether the motion advantage and its proposed attentional mechanisms extend to individuals with DP. Specifically, we examined the effects of facial motion on both familiar (Experiment 1) and unfamiliar face recognition (Experiment 2) in a larger DP sample. This allowed us to address the limited evidence for familiar face recognition and the inconsistent findings reported for unfamiliar face processing in DP. In addition, using eye-tracking, we examined whether facial motion increases attention to internal relative to external facial features in this group, and whether motion maintains attention to the internal features by reducing shifts in visual attention across the face.

Participants with DP were asked to recognise faces presented as either moving video clips or static images while their eye movements were recorded. Performance was compared against an age-matched control group. It was hypothesised that the DP group would show reduced recognition accuracy overall, but that both groups would demonstrate improved performance for moving relative to static faces. In addition, motion was expected to increase attention to the internal facial features and reduce attentional shifts across the face. Finally, any recognition advantage for moving faces was predicted to be associated with this change in visual attention.

## 2. Experiment 1

### 2.1. Method

#### 2.1.1. Design

**Figure 1:**
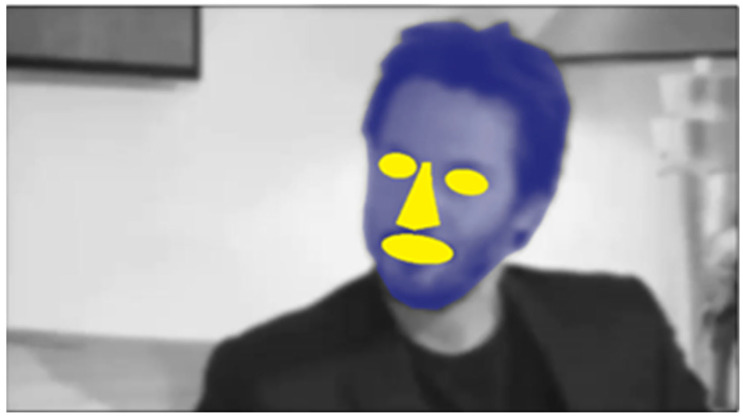
Internal and external interest areas used in Experiments 1 and 2. Note. Internal features (yellow) comprised the eyes, nose, and mouth. The external region was defined as the full face area (blue) with the internal features subtracted.

A 2 × 2 × 2 mixed design was used to assess the impact of motion on familiar face recognition. The first independent variable (IV) was presentation style (moving, static). The second IV, included in the eye-movement analyses only, was interest area (IA), with two levels: internal features (eyes, nose, mouth) and external features (forehead, chin, cheeks, hair; see Figure 1). These first two IVs were manipulated within participants. For the DP analysis, group (DP vs. aged-matched control) was included as an additional between-participants IV.

The first dependent variable (DV) was recognition accuracy, defined as the proportion of correct responses for faces reported as familiar. Eye-movement measures included proportional dwell time (the proportion of the total trial time spent within each IA), proportional fixation count (the proportion of fixations in a trial that fell within each IA) and fixation shifting count (the number of visual shifts between key facial features, including the left eye, right eye, nose, mouth and external regions). Eye-movement analyses focused on correct trials only, as the primary aim was to examine attentional allocation associated with successful face recognition. Consequently, fewer trials contributed to the DP analyses because of lower overall recognition accuracy in this group. However, exploratory analyses including incorrect trials revealed a broadly similar overall pattern of findings.

#### 2.1.2. Participants

**Control samples.** Fifty-one participants (42 female, 9 male) aged between 18 and 61 (M = 26.02, SD = 9.94) were recruited using opportunity sampling. All reported normal or corrected-to-normal vision and no difficulties with face recognition. Exclusion criteria included a history of neurological illness or injury, schizophrenia, autism spectrum disorder (ASD), or any cognitive impairment affecting task performance. Due to technical issues, data from two participants were excluded, resulting in a final sample of 49 (40 female; aged 18–70 years, M = 31.02, SD = 16) for the initial replication analysis. Sixteen control participants were then selected from the larger control sample to form a broadly age-comparable control group for comparisons with the DP group. Control participants aged 35 years and above were selected to align with the age profile of the DP sample (11 female, aged 35–61, M = 52.31, SD = 13.28). An independent-samples *t*-test confirmed that the groups did not significantly differ in age.

**DP sample.** Fourteen individuals with developmental prosopagnosia (10 female, 4 male; aged 40–75 years, M = 58.43, SD = 10.95) were recruited via a dedicated website. During an initial online screening, participants completed the 20-Item Prosopagnosia Index (PI20 [43]), a self-report measure of prosopagnosic traits. All participants scored between 65 and 100, indicating mild to severe prosopagnosia [43]. All also reported severe, lifelong difficulties with face recognition and no history of neurological illness, ASD, or visual or cognitive impairment.

DP status was subsequently confirmed using a standard screening battery employed in previous research [44,45]. Participants completed tests of unfamiliar face recognition (Cambridge Face Memory Test [46]), familiar face recognition (Famous Faces Test [45]), and face perception (Cambridge Face Perception Test [45]). All participants performed at least 2 SDs below normative means on two or more tasks, consistent with previously used diagnostic criteria [47,48]. Lower-level visual functioning (BORB subtests: size match, length match, position of gap, and orientation match [49]; Leuven Perceptual Organisation Screening Test [50]), object recognition (BORB Object Decision [49]), and general intellectual functioning (Test of Premorbid Functioning [51]) were within the typical range. Autistic traits (Autism Spectrum Quotient [52]) were below the clinical cut-off in all cases. All participants provided written informed consent.

#### 2.1.3. Materials

Stimuli consisted of 60 famous faces (30 male, 30 female), including actors, television personalities, politicians, and sports figures. Moving clips were extracted from television interviews (via YouTube) and primarily contained non-rigid motion (e.g., speech and expressions), with some rigid head movement. Static stimuli were single freeze-frames from the same clips showing a clear view of the face. All stimuli were presented for 2 s.

To reduce ceiling effects and match our previous research conditions [30], stimuli were converted to greyscale and blurred using GIMP. Images were 840 × 480 pixels and presented centrally on a 21-inch CRT monitor (1024 × 768 resolution; 160 Hz refresh rate), with a fixed viewing distance of 65 cm.

The experiment was programmed using SR Research Experiment Builder. Eye movements were recorded monocularly using an EyeLink 2 system (500 Hz sampling rate). A 9-point calibration was completed on the dominant eye prior to testing.

#### 2.1.4. Procedure

The 60 faces were presented in two blocks of 30 (15 male, 15 female). Each participant viewed one block in a moving format and the other in a static format. Presentation style and block order were counterbalanced across participants, resulting in four task configurations. Face order was randomised within each block.

Each trial began with a central fixation cross (500 ms) and an automated drift correction procedure. Recalibration was performed if the participant’s gaze deviated by more than 1° from the central fixation. Faces were then presented centrally for 2000 ms, matching the presentation duration used in our previous work [30] to maintain consistency across experimental paradigms.

Following each trial, participants verbally identified the face by name or unique semantic information that could not reasonably apply to another individual. For example, ‘Keanu Reeves’ or ‘played John Wick’ would constitute acceptable responses for Keanu Reeves, whereas broader responses such as ‘an actor’ or ‘appeared in The Matrix’ would not. Where responses were considered ambiguous, participants were prompted to provide additional identifying information. Responses were self-paced; participants pressed a key to continue. After completing both blocks, participants judged the familiarity of each identity on a scale of zero (unfamiliar) to three (highly familiar). Participants were instructed to base their judgments on how familiar they were with each individual prior to completing the experiment. Trials involving unfamiliar faces (defined as a score of zero on the familiarity check) were excluded from the analysis on a participant-by-participant basis. Between 0 and 29 trials were excluded for control participants (M = 8.69, SD = 7.08) and 0–33 trials for the DP group (M = 9.93, SD = 9.98). No trials were excluded on the basis of eye-tracking data quality.

### 2.2. Results

Unless otherwise stated, all variables were normally distributed, as assessed using a Shapiro–Wilk test. This applies to all analyses reported in Experiments 1 and 2.

#### 2.2.1. Motion Effects in the Replication Sample

**Behavioural analyses.** A paired-samples *t*-test revealed a significant effect of presentation style on recognition accuracy, *t*(48) = −5.07, *p* < 0.001, *d* = 0.72, with performance higher for moving (M = 74.84, SD = 15.02) compared to static (M = 67.76, SD = 16.2) faces (mean difference = 7.08%, 95% CI [4.27, 9.89]). Within the sample, 37 participants demonstrated greater accuracy for moving faces (i.e., a motion advantage), 10 demonstrated higher accuracy for static faces, and two performed at equal levels for both presentation styles.

**Eye-tracking analyses:** Descriptive statistics (means and standard deviations) for proportional dwell time and fixation count to internal and external IAs are presented in Table 1. The data were analysed using 2 (presentation style: moving, static) × 2 (IA: internal, external) repeated-measures ANOVAs. Any violation of the sphericity assumption was adjusted for using the Greenhouse–Geisser correction [53]. Bonferroni corrections [54] were applied to all follow-up pairwise comparisons and simple effects analyses across Experiments 1 and 2 to mitigate the risk of type I error. The results revealed a significant main effect of IA for both dwell time, *F*(1, 48) = 39.90, *p* < 0.001, ω^2^ = 0.44, and fixation count, *F*(1, 48) = 41.22, *p* < 0.001, ω^2^ = 0.44, indicating greater attention to the internal IA overall. There was no main effect of presentation style on dwell time, *F*(1, 48) = 1.83, *p* = 0.18, ω^2^ < 0.01, but there was a significant effect on fixation count, *F*(1, 48) = 6.81, *p* = 0.01, ω^2^ = 0.04, with more fixations falling on the face (as opposed to the background) on moving trials. Critically, these effects were qualified by a significant interaction between presentation style and IA for both dwell time, *F*(1, 48) = 18.45, *p* < 0.001, ω^2^ = 0.23, and fixation count, *F*(1, 48) = 140.11, *p* < 0.001, ω^2^ = 0.68. Simple effects analyses showed that participants directed a greater proportion of fixations to the internal IA in the moving condition relative to the static condition (mean difference =13.67%, 95% CI [10.42, 16.93]), whereas greater attention was directed to the external IA in the static condition compared with the moving condition, (mean difference = 11.35%, 95% CI [8.3, 14.41]). A similar pattern was observed for dwell time.

To examine whether this effect was driven by attention to one specific internal feature, proportional dwell time and fixation count directed to the eyes, nose, and mouth were analysed using 2 (presentation style: moving, static) × 3 (IA: eyes, nose, mouth) repeated-measures ANOVA. There were significant main effects of presentation style for both dwell time, *F*(1, 48) = 12.30, *p* = 0.001, ω^2^ = 0.03, and fixation count, *F*(1, 48) = 133.66, *p* < 0.001, ω^2^ = 0.2, indicating greater attention to internal features during moving relative to static trials. There were also significant main effects of IA for dwell time, *F*(2, 96) = 10.62, *p* < 0.001, ω^2^ = 0.15 and fixation count, *F*(2, 96) = 12.17, *p* < 0.001, ω^2^ = 0.16, with more attention directed to the nose than the mouth (*p* < 0.001; all other *p*s > 0.05). However, there was no significant interaction between presentation style and IA for either dwell time, *F*(2, 96) = 0.65, *p* = 0.52, ω^2^ < 0.001, or fixation count, *F*(2, 96) = 0.92, *p* = 0.40, ω^2^ < 0.001, indicating that motion increased attention across all internal features.

To assess whether motion also influenced the maintenance of attention, fixation shifting between IAs was analysed. A fixation shift was defined as a change in gaze from one IA (left eye, right eye, nose, mouth, external) to another on consecutive fixations. Participants showed significantly fewer fixation shifts when viewing moving (M = 3.03, SD = 0.79) compared to static faces (M = 3.54, SD = 0.79), *t*(49) = 6.35, *p* < 0.001, *d* = 0.91, indicating reduced visual sampling across IAs in the moving condition (mean difference = 0.51 shifts per trial, 95% CI [0.34, 0.67]).

#### 2.2.2. Comparison of DP and Age-Matched Controls

**Behavioural analyses.** A 2 (presentation style: moving, static) × 2 (group: DP, control) mixed ANOVA revealed a significant main effect of presentation style on recognition accuracy, *F*(1, 28) = 5.04, *p* < 0.05, ω^2^ = 0.04, with higher accuracy for moving (M = 59.88%) relative to static faces (M = 54.54%; mean difference = 5.34%, 95% CI [0.46, 10.22]). There was also a significant main effect of group, *F*(1, 28) = 48.58, *p* < 0.001, ω^2^ = 0.53, with control participants (M = 75.96%) outperforming the DP group (M = 38.47%; mean difference = 37.49%, 95% CI [26.48, 48.51]). The interaction between presentation style and group was not significant, *F*(1, 28) = 0.04, *p* = 0.84, ω^2^ < 0.01. This indicated no clear evidence of differential motion benefits across groups, although subtle group differences may not have been detectable given the sample size. In the DP group, eight participants demonstrated greater accuracy for moving faces, and six showed higher accuracy for static faces. In the control group, 11 participants showed a motion advantage, four showed higher accuracy for static faces, and one performed equally across conditions.

**Eye-tracking analyses.** Proportional dwell time and fixation count directed to internal and external IAs were analysed using 2 (presentation style: moving, static) × 2 (IA: internal, external) × 2 (group: DP, control) mixed ANOVAs. Descriptive statistics are presented in Table 2. There was a significant main effect of IA for both dwell time, *F*(1, 28) = 75.88, *p* < 0.001, ω^2^ = 0.72, and fixation count, *F*(1, 28) = 75.88, *p* < 0.001, ω^2^ = 0.75, indicating greater attention to the internal IA overall. There was also a significant main effect of presentation style on fixation count, *F*(1, 28) = 17.85, *p* < 0.001, ω^2^ = 0.08, but not on dwell time, *F*(1, 28) = 2.73, *p* = 0.11, ω^2^ = 0.01, indicating more fixations were directed to the face versus the background in the moving condition. Critically, a significant interaction between presentation style and IA was observed for both dwell time, *F*(1, 28) = 5.41, *p* < 0.05, ω^2^ = 0.12, and fixation count, *F*(1, 28) = 68.35, *p* < 0.001, ω^2^ = 0.66. Simple main effects analyses showed that participants directed a greater proportion of fixations (*p* < 0.001, mean difference = 12.51%, 95% CI [9.61, 15.41]), but not dwell time (*p* = 0.07) to the internal IA when faces were presented in motion, whereas a greater proportion of dwell time (*p* < 0.01, mean difference = 4%, 95% CI [1.07, 6.93]) and fixations (*p* < 0.001, mean difference = 10.16%, 95% CI [7.32, 13]) were directed to the external IA in the static condition. No interactions involving group were significant (all *p*’s > 0.10), indicating similar patterns across DP and control participants.

**Figure 2:**
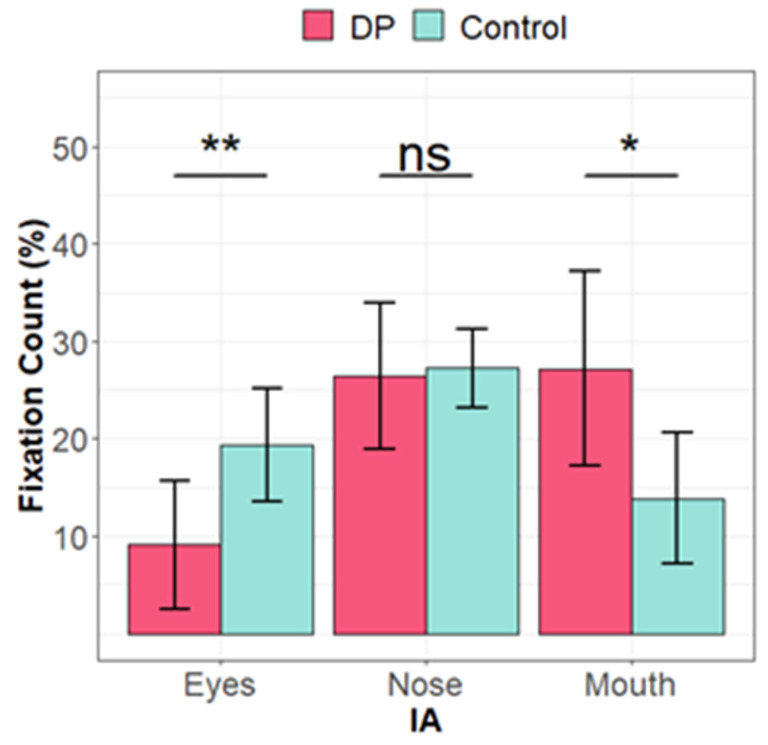
Interaction between group and IA (eyes, nose, and mouth) on proportional fixation count. **Note.** Error bars represent 95% confidence intervals (applies to all figures). Asterisks denote significance: * *p* < 0.05, ** *p* < 0.01, ns: not significant.

To examine whether the increased proportion of fixations to the internal features during moving trials relative to static trials was driven by attention to a specific feature, proportional fixation count directed to the eyes, nose, and mouth was analysed using a 2 (presentation style: moving, static) × 3 (IA: eyes, nose, mouth) × 2 (group: DP, control) mixed ANOVA. There were significant main effects of presentation style, *F*(1, 28) = 78.25, *p* < 0.001, ω^2^ = 0.32, and IA, *F*(2, 56) = 7.07, *p* < 0.01, ω^2^ = 0.17. There was also a significant interaction between IA and group, *F*(2, 56) = 6.26, *p* < 0.01, ω^2^ = 0.15, reflecting reduced attention to the eyes (mean difference = 12.75, 95% CI [3.76, 21.73]) and increased attention to the mouth (mean difference = 16.33, 95% CI [3.68, 28.97]) in the DP group relative to the control group (see Figure 2). However, there was no interaction between presentation style and IA, *F*(2, 56) = 0.03, *p* = 0.98, ω^2^ < 0.01, indicating that the effect of motion did not differ across the internal features.

To assess whether motion also influenced the maintenance of attention, fixation-shifting counts were analysed using a 2 (presentation style: moving, static) × 2 (group: DP, control) mixed ANOVA. The results revealed a significant main effect of presentation style, *F*(1, 28) = 33.22, *p* < 0.001, ω^2^ = 0.12, with fewer shifts for moving relative to static faces. The main effect of group, *F*(1, 28) = 0.13, *p* = 0.72, and the interaction, *F*(1, 28) = 0.01, *p* = 0.95, were not significant.

### 2.3. Discussion

The results of this experiment demonstrate a clear motion advantage for familiar face recognition, with both groups showing greater accuracy for moving relative to static faces. This finding is consistent with previous research indicating that motion facilitates familiar face recognition [12] and extends this effect to individuals with DP [10]. As expected, the DP group showed reduced overall recognition accuracy, in line with established impairments in familiar face recognition [55]. However, the magnitude of the motion advantage did not differ significantly between groups, suggesting that individuals with DP may benefit from motion in a manner similar to typical recognisers when recognising familiar faces.

Eye-tracking findings indicated that motion influenced visual attention in both groups. Specifically, participants directed a greater proportion of attention to the internal facial features and showed fewer attentional shifts across the face when viewing moving faces than when viewing static faces. These findings suggest that motion both attracts attention to, and maintains attention on, identity-relevant regions of familiar faces, consistent with the assumptions of the social signals hypothesis [20].

Importantly, similar attentional effects were observed in the DP group, suggesting that motion may support recognition by influencing face-scanning strategies. However, group differences in fixation patterns were evident, with individuals with DP directing less attention to the eye region and more attention to the mouth than controls, consistent with previous research using static images [25,56]. Given the diagnostic importance of the eye region for identity recognition [57,58], this atypical allocation of attention may contribute to their reduced overall performance.

Taken together, these findings suggest that motion may facilitate familiar face recognition, at least in part, by influencing attentional allocation in both typical and DP populations. However, given that motion effects are less consistent in unfamiliar face processing (e.g., [7,9]), and evidence for a motion advantage in DP is even more limited and mixed, with studies typically based on very small samples [5,40,41,42], it is important to determine whether similar mechanisms operate during the learning and recognition of unfamiliar identities. This is addressed in Experiment 2.

## 3. Experiment 2

### 3.1. Method

#### 3.1.1. Design

A 2 × 2 × 2 mixed design was used to assess the impact of non-rigid motion on unfamiliar face recognition. The first IV was face presentation style during encoding (moving vs. static). The second IV, included in the eye-movement analyses only, was interest area (IA), with two levels: internal features (eyes, nose, mouth) and external features. Both were manipulated within participants. For the DP analysis, group (DP vs. age-matched control) was included as an additional between-participants IV.

The behavioural DVs were recognition accuracy and RT. Accuracy was operationalised using nonparametric signal-detection measures A’ (sensitivity) and B’’ (response bias), calculated from hit and false-alarm rates [59]. Higher A’ values indicate better discrimination (chance = 0.5), while B’’ reflects response bias, with negative values indicating a bias towards ‘old’ responses and positive values indicating a bias towards ‘new’ responses. As in Experiment 1, eye-movement DVs were proportional dwell time, proportional fixation count and fixation shifting count.

#### 3.1.2. Participants

Participants were the same 51 controls and 14 individuals with DP who completed Experiment 1. There were no technical issues, so all 51 control participants were included in the replication analysis.

#### 3.1.3. Materials

For the learning phase, 20 full-colour videos were selected from the Amsterdam Dynamic Facial Expressions Set [60]. Each video featured a single actor of Northern European ancestry (10 male, 10 female), shown from the shoulders upwards. A moving and static version of each face was created from the 20 videos. Moving stimuli began with a neutral expression, then transitioned to ‘joy’, presented directly to the camera at a face-forward (0°) angle. Static stimuli were created by extracting the final frame from each video using Windows Movie Maker, resulting in a still image of a happy ‘joy’ expression. For both presentation styles, stimuli were displayed for 2 s.

During the recognition phase, participants viewed 40 full-colour facial images depicting the head and shoulders of a single individual of Northern European ancestry. Target stimuli consisted of the 20 identities presented during the learning phase. An additional 20 identities (10 male, 10 female) from the Radboud Faces Database [61] served as distractors. Images were edited using GIMP to minimise differences in lighting, background, and feature alignment across databases. All recognition-phase stimuli displayed neutral expressions to ensure that the task measured identity recognition rather than picture matching [62]. Although participants in the moving condition were exposed to a brief neutral expression during learning (neutral-to-joy transitions), our previous work [30] directly assessed the impact of this methodological factor on performance and found no difference in recognition performance or the magnitude of the motion advantage, suggesting that it is unlikely to influence the results here. All recognition-phase stimuli were presented in static, consistent with evidence that motion is more critical during learning than recognition of unfamiliar faces [6].

Across both phases of the experiment, stimuli were presented at 750 × 570 pixels and eye-tracking data were collected using the same equipment and calibration procedures as described in Experiment 1.

#### 3.1.4. Procedure

During the learning phase, participants viewed 20 faces (10 static, 10 moving) presented sequentially in a random order. They were instructed to learn the faces in preparation for a subsequent recognition task. Facial identities were divided into two sets, with presentation style (static or moving) counterbalanced across two experiment versions, so that each identity appeared equally often in the static and moving conditions across participants.

The recognition phase followed immediately after learning. Participants viewed 40 stimuli (20 targets, 20 distractors) presented in a single randomised block. For each trial, participants indicated whether the face was ‘old’ or ‘new’ using a keyboard response (m or z). Although no time limit was imposed, participants were instructed to respond as quickly and accurately as possible. Each stimulus remained on screen until a response was made.

All stimuli were presented centrally. Prior to the presentation of each face, a central fixation cross was shown for 500 ms, followed by an automated drift correction procedure. Recalibration was performed if gaze deviated by more than 1° from fixation. No trials were excluded on the basis of eye-tracking data quality.

### 3.2. Results

#### 3.2.1. Motion Effects in the Replication Sample

**Behavioural analyses.** Sensitivity and response bias measures violated assumptions of normality; therefore, nonparametric analyses were conducted. Wilcoxon signed-ranks tests revealed a significant effect of presentation style on sensitivity, *Z* = −4.46, *p* < 0.001, with higher accuracy for faces learned in motion (M = 0.9, SD = 0.07) compared to static faces (M = 0.86, SD = 0.1; median difference = 0.05, 95% CI [0.03, 0.07]). A significant effect was also observed for response bias, *Z* = −3.07, *p* < 0.01, with more conservative responding for static faces (M = 0.36, SD = 0.42) than moving faces (M = 0.23, SD = 0.46; median difference = 0.12, 95% CI [0.04, 0.19]). Most participants (*n* = 35) showed greater accuracy for faces learned in motion, eight favoured static faces and eight performed equally for both presentation styles.

RTs were calculated for correct trials only. A paired-samples *t*-test showed a significant effect of presentation style, *t*(50) = 3.05, *p* = 0.004, *d* = 0.43, with faster responses for faces learned in motion (M = 1570.28 ms, SD = 889.25) compared to static faces (M = 1694.17 ms, SD = 1015.03; mean difference = 123.88 ms, 95% CI [42.22, 205.54]).

**Eye-tracking analyses:** Eye movements were analysed separately for the learning and recognition phases. Analyses focused on correctly recognised trials only (i.e., at learning: faces subsequently correctly recognised at test; at recognition: hits).

Descriptive statistics for proportional dwell time and fixation count during the *learning* *phase* are presented in Table 3. A 2 (presentation style: moving vs. static) × 2 (interest area: internal vs. external) repeated-measures ANOVA revealed a significant main effect of interest area for both dwell time, *F*(1, 50) = 416, *p* < 0.001, ω^2^ = 0.89, and fixation count, *F*(1, 50) = 343.85, *p* < 0.001, ω^2^ = 0.87, with greater attention directed to internal features. A significant main effect of presentation style was found for dwell time, *F*(1, 50) = 29.27, *p* < 0.001, ω^2^ = 0.14, but not fixation count, with a greater proportion of dwell time directed to the face, relative to the background, during moving trials. A significant interaction between presentation style and interest area was observed for both dwell time, *F*(1, 50) = 8.88, *p* < 0.01, ω^2^ = 0.13, and fixation count, *F*(1, 50) = 11.36, *p* = 0.001, ω^2^ = 0.16. Simple main effects revealed that participants allocated more fixations to the internal features when faces were learned in motion (mean difference = 3.03%, 95% CI [0.69, 5.37]), and more fixations to the external features when learned from static images (mean difference = 4.01%, 95% CI [1.6, 6.43]). A similar pattern was observed for dwell time.

To examine whether the interaction was driven by attention to one specific internal feature, dwell time and fixation count were analysed using a 2 (presentation style: moving vs. static) × 3 (interest area: eyes, nose, mouth) repeated-measures ANOVA. The results revealed a significant main effect of presentation style on both dwell time, *F*(1, 50) = 6.76, *p* < 0.05, ω^2^ = 0.02, and fixation count, *F*(1, 50) = 11.20, *p* < 0.01, ω^2^ = 0.02, with greater attention to internal features for faces learned in motion. A significant main effect of IA was also observed for dwell time, *F*(2, 100) = 15.72, *p* < 0.001, ω^2^ = 0.21, and fixation count, *F*(2, 100) = 15.97, *p* < 0.001, ω^2^ = 0.21. Bonferroni-corrected comparisons showed greater dwell time to the eyes than the nose (*p* < 0.01) and mouth (*p* < 0.001), and more fixations to the eyes and nose than the mouth (*p* < 0.001). Finally, there was no significant interaction between presentation style and interest area for dwell time, *F*(2, 100) = 1.24, *p* = 0.29, ω^2^ < 0.01, or fixation count, *F*(2, 100) = 0.31, *p* = 0.73, ω^2^ < 0.01, indicating that the effect of motion was similar across the internal features.

Descriptive statistics for proportional dwell time and fixation count during the *recognition phase* are presented in Table 4. A 2 (presentation style at learning) × 2 (interest area) ANOVA revealed a significant main effect of interest area on dwell time, *F*(1, 50) = 504.76, *p* < 0.001, ω^2^ = 0.91, and fixation count, *F*(1, 50) = 457.5, *p* < 0.001, ω^2^ = 0.90, with greater attention directed to the internal features. No significant effects of presentation style or interactions were found, indicating no carry-over effect of learning condition on gaze patterns at test.

Fixation shifts were calculated using the same technique employed in Experiment 1. Wilcoxon signed-rank tests revealed that more fixation shifts occurred for faces learned in a static format compared to those learned in a moving format during both learning, *Z* = −3.06, *p* = 0.002 (median difference = 0.45 fixation shifts per trial, 95% CI [0.15, 0.75]), and recognition, *Z* = −2.22, *p* = 0.03 (mean difference = 0.17 fixation shifts per trial, 95% CI [0.01, 0.26]). This suggests that static faces required greater visual sampling, whereas motion was associated with reduced attentional shifts.

#### 3.2.2. Comparison of DP and Age-Matched Controls

**Behavioural analyses.** Sensitivity (A’) was analysed using a 2 (presentation style: moving vs. static) × 2 (group: DP vs. control) mixed ANOVA. There were significant main effects of presentation style, *F*(1, 28) = 13.62, *p* < 0.01, ω^2^ = 0.10, and group, *F*(1, 28) = 7.00, *p* = 0.01, ω^2^ = 0.09, reflecting greater accuracy in the moving-face condition relative to static (mean difference = 0.04, 95% CI [0.02, 0.06]) and in the control group relative to the DP group (mean difference = 0.05, 95% CI [0.01, 0.09]), respectively. The interaction was not significant, *F*(1, 28) = 1.88, *p* = 0.18, ω^2^ < 0.01, providing no evidence that the effect of motion differed between the DP and control groups. However, this finding should be treated with caution due to the small sample size. Within the DP group, 10 participants showed a motion advantage, three a static advantage, and one showed no difference. In the control group, eight showed a motion advantage, two a static advantage, and six showed no difference.

The same analysis for the B’’ data revealed no significant effect of presentation style, *F*(1, 28) = 2.19, *p* = 0.15, ω^2^ = 0.01, or group, *F*(1, 28) = 1.78, *p* = 0.19, ω^2^ = 0.01, and no significant interaction between presentation style and group, *F*(1, 28) = 0.08, *p* = 0.78, ω^2^ < 0.01.

RTs (log-transformed) for correct responses revealed a main effect of group, *F*(1, 28) = 33.08, *p* < 0.001, ω^2^ = 0.30, with slower responses in the DP group (M = 2735.53 ms, SD = 1699.5) compared to the control (M = 1293.09 ms, SD = 372.67). There was no effect of presentation style, *F*(1, 28) = 2.84, *p* = 0.10, ω^2^ < 0.01, and no interaction, *F*(1, 28) = 0.08, *p* = 78, ω^2^ < 0.01.

**Eye-tracking analyses.** Descriptive statistics for dwell time and fixation count across interest areas during the learning phase are presented in Table 5.

A 2 (presentation style) × 2 (interest area: internal vs. external) × 2 (group) ANOVA showed a main effect of interest area for both dwell time, *F*(1, 28) = 128.97, *p* < 0.001, ω^2^ = 0.82, and fixation count, *F*(1, 28) = 146.70, *p* < 0.001, ω^2^ = 0.84, with greater attention directed to the internal features. A main effect of presentation style was also observed for dwell time only, *F*(1, 28) = 69.72, *p* < 0.001, ω^2^ = 0.30, with greater overall face-directed dwell time (i.e., proportion of viewing time allocated to the face relative to the background) in the static condition. Although statistically significant, this effect reflected only a small absolute difference in viewing behaviour, with participants directing slightly more attention towards the background in the moving condition.

**Figure 3:**
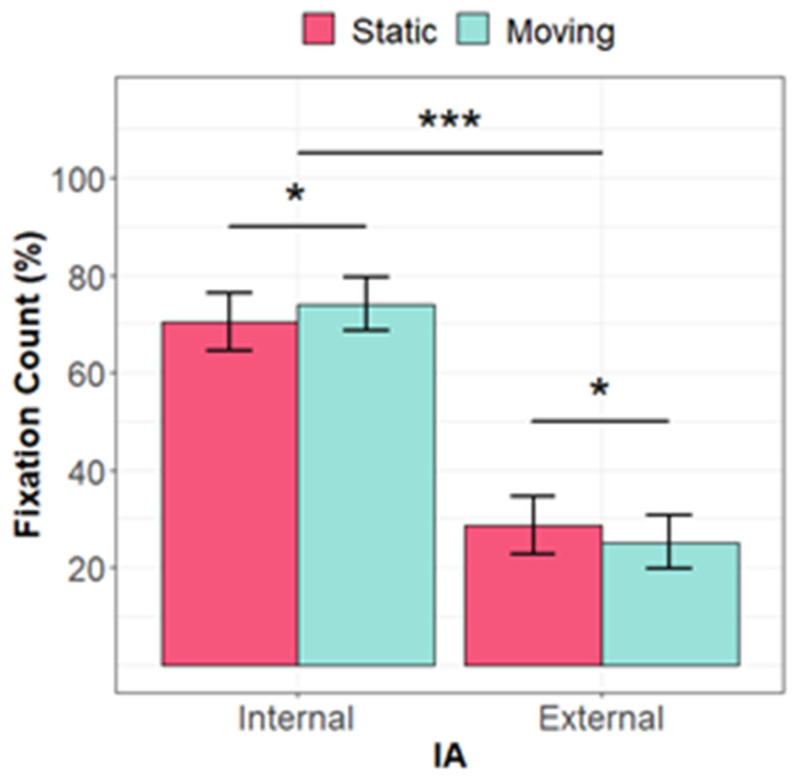
Interaction between presentation style and IA (internal and external features) on proportional fixation count during face learning; * *p* < 0.05, *** *p* < 0.001.

**Figure 4:**
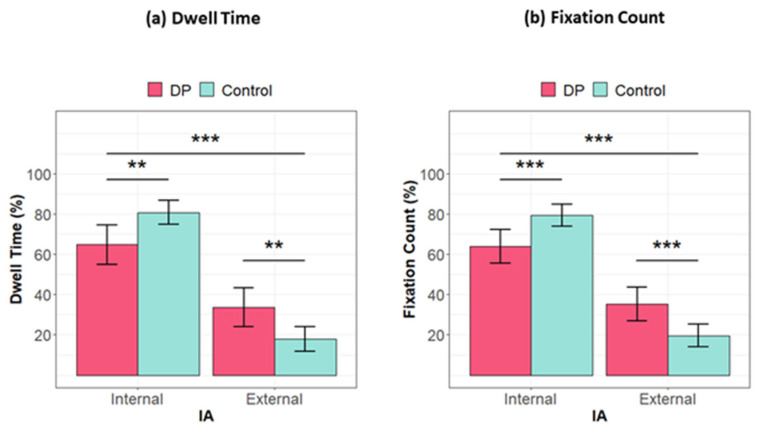
Interaction between group and IA (internal and external features) on proportional dwell time (**a**) and fixation count (**b**) during face learning; ** *p* < 0.01, *** *p* < 0.001.

A significant interaction between presentation style and interest area was observed for fixation count, *F*(1, 28) = 5.86, *p* < 0.05, ω^2^ = 0.14 (Figure 3), but not dwell time, indicating that a greater proportion of fixations was directed to the internal features in the moving condition relative to the static condition (mean difference = 3.62%, 95% CI [0.575, 6.67]). A larger interaction between interest area and group was also found for both dwell time, *F*(1, 28) = 14.73, *p* < 0.01, ω^2^ = 0.32, and fixation count, *F*(1, 28) = 18.11, *p* < 0.001, ω^2^ = 0.37 (Figure 4). Participants in the control group directed relatively more fixations to the internal features than the DP group (mean difference = 15.43%, 95% CI [7.67, 23.19]), whereas the DP group directed relatively more fixations to the external features than the control group (mean difference = 15.69%, 95% CI [8.41, 22.97]). A similar pattern was observed for dwell time. All other main and interaction effects were non-significant (*p*s > 0.05).

**Figure 5:**
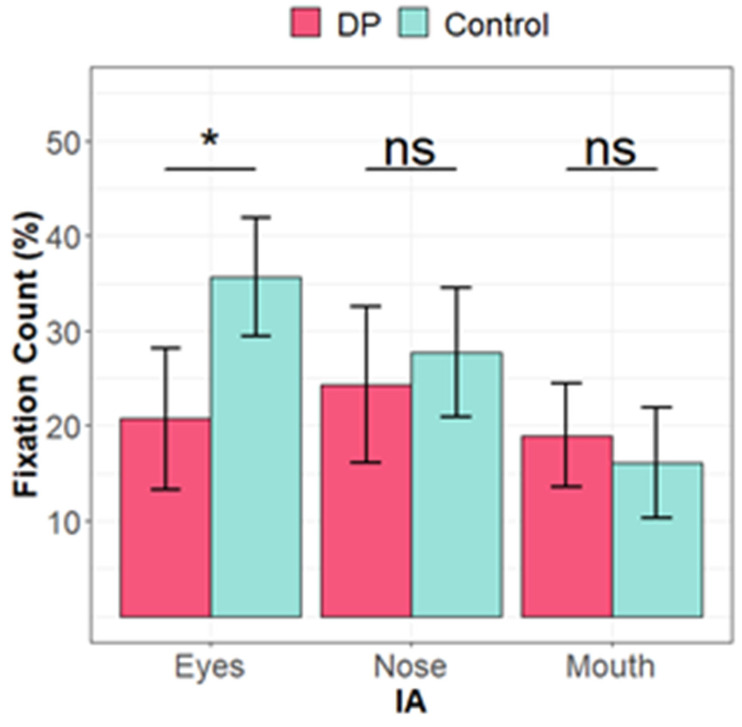
Interaction between group and IA (eyes, nose, and mouth) on proportional fixation count during face learning; * *p* < 0.05, ns: not significant.

A 2 (presentation style) × 3 (interest area: eyes, nose and mouth) × 2 (group) ANOVA was performed to determine if the increased proportion of fixations directed to the internal features during moving trials relative to static could be attributed to attention to one specific feature. The ANOVA revealed a significant main effect of presentation style on fixation count, *F*(1, 28) = 5.93, *p* < 0.05, ω^2^ = 0.02, with a greater proportion of fixations directed to the internal features when faces were presented in motion. There was also a significant main effect of IA, *F*(2, 56) = 7.04, *p* < 0.01, ω^2^ = 0.16, with more attention directed to the nose than the mouth. An interaction between interest area and group, *F*(2, 56) = 4.52, *p* < 0.05, ω^2^ = 0.10, reflected greater fixation to the eye region in the control group relative to the DP group (mean difference = 16.29%, 95% CI [5.81, 26.77], Figure 5). No other effects were significant.

**Figure 6:**
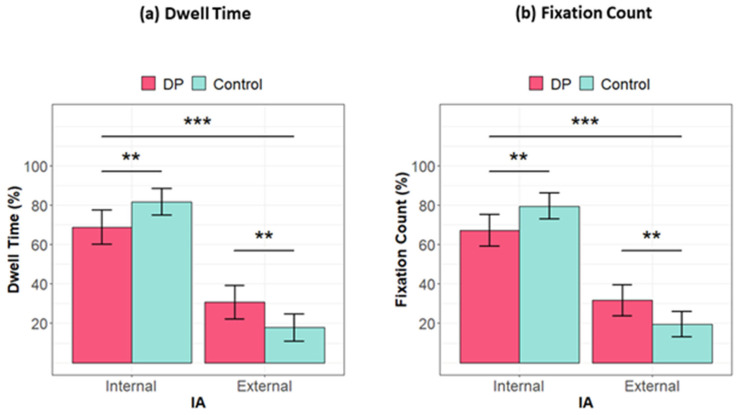
Interaction between group and IA (internal and external features) on proportional dwell time (**a**) and fixation count (**b**) during face recognition; ** *p* < 0.01, *** *p* < 0.001.

Descriptive statistics for proportional dwell time and fixation count directed to the internal and external features during the recognition phase are presented in Table 6. A three-way ANOVA revealed a main effect of interest area on dwell time, *F*(1, 28) = 171.93, *p* < 0.001, ω^2^ = 0.85, and fixation count, *F*(1, 28) = 178.84, *p* < 0.001, ω^2^ = 0.86, with greater attention to internal features. This was qualified by an interaction with group (dwell time: *F*(1, 28) = 10.94, *p* < 0.01, ω^2^ = 0.25; fixation count: *F*(1, 28) = 11.88, *p* < 0.01, ω^2^ = 0.27), again reflecting reduced internal feature and increased external feature attention in the DP group. For fixation count, the control group directed a greater proportion of fixations to the internal features than the DP group (mean difference = 12.84%, 95% CI [4.87, 20.82]); a similar pattern was observed for dwell time (see Figure 6).

Fixation-shifting counts at learning and test were analysed using 2 (presentation style) × 2 (group) ANOVA. Results revealed a main effect of presentation style during face learning, *F*(1, 28) = 4.24, *p* < 0.05, ω^2^ = 0.01, with fewer fixation shifts for faces learned in motion than for those learned in a static form (mean difference = 0.39, 95% CI [0.002, 0.79]. No group differences or interactions were observed (*p*s > 0.33). During recognition, there was a main effect of group, *F*(1, 28) = 9.12, *p* < 0.01, ω^2^ = 0.12, with more fixation shifts in the DP group compared to the control group (mean difference = 3.36, 95% CI [1.08, 5.62]. No other effects were significant.

### 3.3. Discussion

The results of Experiment 2 indicate that non-rigid motion facilitates the learning of unfamiliar faces, with participants showing faster and more accurate recognition for faces learned in motion compared to static. This is consistent with previous research demonstrating a motion advantage during unfamiliar face learning [6,7,9].

Eye-tracking results showed that motion influenced attentional allocation during face learning. Specifically, participants directed a greater proportion of attention to the internal facial features and showed fewer fixation shifts when learning faces in motion compared to static. These findings suggest that motion is associated with increased attention towards identity-relevant regions and reduced attentional shifting across the face, consistent with the attentional assumptions of the social signals hypothesis [20].

The results from the DP group broadly mirrored those of the control group. Individuals with DP showed reduced recognition accuracy overall, consistent with established impairments in processing unfamiliar faces [32,47]. However, both groups demonstrated improved performance for faces learned in motion, suggesting that motion can support unfamiliar face learning in DP. As in typical recognisers, motion increased attention to the internal features and reduced fixation shifting during learning, indicating that similar attentional mechanisms may operate in both groups. Group differences in scanning behaviour were also evident, with individuals with DP directing less attention to the internal features, particularly the eye region, than controls, consistent with previous research [25]. This atypical allocation of attention may contribute to their reduced recognition performance.

It is important to note, however, that recognition stimuli were drawn from two different face databases, with targets from the ADFES database and distractors from the Radboud. Although images were standardised and there was no evidence of a systematic response bias in the b′ analyses, database-specific differences may nevertheless have introduced additional variability into recognition performance. As such, the findings should be interpreted with some caution until replicated using stimuli drawn from a single database. Nevertheless, the consistency of the motion advantage across behavioural and eye-tracking measures suggests that the observed effects are unlikely to be solely attributable to database differences.

Taken together, these findings suggest that motion facilitates the learning of unfamiliar faces and is associated with changes in attentional allocation during encoding. The results are also consistent with accounts that emphasise the role of motion in enhancing the structural representation of unfamiliar faces. The relative contribution of these attentional and representational mechanisms is considered further in the general discussion.

## 4. General Discussion

The present study examined whether the motion advantage in face recognition is associated with changes in visual attention, and whether these effects extend to individuals with developmental prosopagnosia (DP). To our knowledge, this is the first time eye-movement differences for moving and static faces have been assessed in individuals with DP. Across two experiments, facial motion improved recognition performance and altered the way participants sampled information from the face. In both familiar- and unfamiliar face tasks, motion increased attention to internal facial features, replicating, for the first time, the findings of Butcher et al. [30] and extending them to reveal that facial motion was also associated with reduced attentional shifting across the face. These effects were observed in both typical recognisers and individuals with DP, although the DP group showed lower overall recognition accuracy and differences in the distribution of attention across internal features.

At a behavioural level, the findings indicate that motion facilitated both familiar face recognition and unfamiliar face learning. Participants were more accurate when recognising famous faces in motion than when recognising the same faces in static form (Experiment 1), and faster and more accurate when recognising unfamiliar faces that had been learned in non-rigid motion (Experiment 2). These findings are consistent with previous research showing that motion can facilitate familiar face recognition, particularly under non-optimal viewing conditions, and can also benefit unfamiliar face learning, although the latter effect has been less consistently observed in the literature [6,7,12].

That said, the presence of substantial individual differences across both experiments and within both the DP and typical recogniser groups suggests that the motion advantage is not universal. Some participants showed little benefit from motion, and others performed better in the static condition. This variability is consistent with earlier reports in both typical and atypical populations, suggesting that motion does not uniformly facilitate recognition. Instead, the contribution of motion depends on characteristics of the observer and the task. This finding necessitates future research into the mechanisms underlying these individual differences in the motion advantage.

Turning now to the eye-tracking findings, support for the idea that facial motion alters attention allocation across the face was established. In both experiments, motion increased the proportion of dwell time and fixations directed to the internal features, replicating our earlier work [10], and revealed for the first time that facial motion also reduces the number of fixation shifts between facial regions. This novel finding may have theoretical implications for accounts such as the social signals hypothesis (SSH [20]), as the observed pattern is broadly consistent with the proposal that facial motion helps direct and maintain attention towards identity-relevant facial regions over time. Importantly, this effect was not restricted to the eye region alone. For instance, in the familiar face task, motion increased attention across the eyes, nose, and mouth, rather than selectively enhancing attention to a single internal feature. This suggests that motion may promote a broader shift towards the internal face region as a whole.

However, the present findings should be interpreted cautiously in relation to the SSH. The dynamic stimuli in Experiment 1 were intentionally naturalistic and therefore contained multiple forms of facial movement, including expressions, speech-related movement, and eye and head movements. As a result, it is not possible to determine whether the observed effects were driven specifically by the social nature of the motion, as proposed by the SSH, or by more general properties of dynamic stimuli, such as increased visual salience. Although Experiment 2 employed more controlled dynamic stimuli, the motion stimuli remained social in nature and lacked a non-social motion comparison condition. Therefore, the present experiments do not provide a direct test of the SSH. Future work should compare different motion types more systematically, including rigid versus non-rigid motion and social versus non-social facial movements.

Additionally, while the combination of increased attention towards the internal features and reduced fixation shifting is consistent with the idea that motion helps attract and maintain attention to identity-relevant facial regions, the present findings do not directly establish that these attentional changes underpin the behavioural motion advantage. Future research should therefore seek to recruit larger samples so that the relationship between motion-related changes in attention and recognition performance can be more reliably examined, including through mediation analyses.

The two experiments also suggest that the contribution of differential attentional allocation to the motion advantage may differ depending on task demands and face familiarity. In the familiar face task, the motion advantage and the associated increased attention to internal features were observed at the point of recognition. This finding is compatible with the supplemental information hypothesis (SIH [13]), insofar as familiar faces may be recognised partly from characteristic movement patterns. In contrast, in the unfamiliar face task, motion influenced attention to internal features during face learning. In this respect, the findings are also consistent with the representation enhancement hypothesis (REH [13]), which proposes that motion supports the formation of a richer structural representation of a face during learning. Taken together, the present findings suggest that the motion advantage is unlikely to be explained by a single mechanism. Instead, attentional, representational, and supplementary cue-based processes may all contribute, with their relative importance varying across tasks and face familiarity.

The findings also have important implications for understanding face processing in DP. Across both experiments, individuals with DP showed reduced recognition accuracy overall, consistent with established impairments in familiar and unfamiliar face recognition. Nevertheless, they benefitted from motion to a similar extent as the control group at the group level. This suggests that dynamic information can support face recognition even when static processing is impaired. Indeed, some individuals with DP displayed poorer performance for static than moving faces, raising the possibility that static and dynamic face recognition may be differentially affected in at least some cases. This fits with accounts proposing partially distinct pathways for processing invariant and changeable facial information [63,64] and suggests that motion may provide access to useful identity-diagnostic information that is less readily available from static images alone.

Although motion influenced attentional allocation in both typical recognisers and individuals with DP, important group differences were also observed. Individuals with DP showed a different distribution of attention across the internal features, with relatively less attention to the eye region and greater attention to the mouth than controls. In Experiment 2, they also directed less attention to the internal features overall during unfamiliar face processing. These findings are in line with previous reports of atypical scan patterns in DP (e.g., [25,56]) and suggest that atypical allocation of attention may at least contribute to their recognition difficulties. Given the diagnostic importance of the eyes for identity recognition [23,24], reduced attention to this region may be especially consequential. The present findings, therefore, raise the possibility that motion may benefit individuals with DP, at least in part, by shifting attention towards more useful regions of the face. This has potential implications for intervention, although any practical application would depend on a better understanding of which kinds of motion are most helpful and for whom, and the functionality of eye-movement differences on moving and static face recognition accuracy in DP.

Several limitations should be acknowledged. First, in Experiment 1, the famous-face stimuli inevitably varied across several dimensions, including viewpoint, lighting, expressive content, and clip quality. Such variability is difficult to avoid when using ecologically valid familiar face videos, and familiar face recognition is generally considered relatively robust to such variation [65,66,67]. Nevertheless, greater experimental control over stimulus properties would enable a more precise examination of the factors contributing to performance differences between static and dynamic conditions.

In Experiment 2, all faces were presented in static format at test. Although this design allowed the effects of motion during learning to be isolated, it also limits conclusions about the role of motion at retrieval. Previous work suggests that motion may be most beneficial during encoding for previously unfamiliar faces [6,68], but the present study cannot rule out the possibility that newly learned dynamic information could also contribute at recognition under some conditions. Future research should therefore manipulate motion independently at learning and test to determine more precisely when motion exerts its strongest effects.

Second, the DP findings should be interpreted with caution, given the relatively small sample size; however, it is worth noting that this sample is larger than those typically reported in previous studies of the motion advantage in DP, reflecting the practical challenges of recruiting this population. DP is a heterogeneous condition, with marked variability in both behavioural performance and face-scanning patterns [44,69,70]. Although the present results were broadly consistent across group-level analyses, a larger sample would allow for a more robust examination of individual differences in the motion advantage and its attentional correlates. This is particularly important given that not all participants benefitted from motion, and some showed the opposite pattern.

An additional consideration relates to how familiarity was operationalised in Experiment 1. Faces were classified as either familiar or unfamiliar, but familiarity is unlikely to be all-or-none. Previous research suggests that the salience of internal features increases as familiarity develops [71,72], and that motion becomes a more useful cue as knowledge of a face’s characteristic movements increases [11]. Treating familiarity as a graded construct may therefore provide a clearer indication of how prior experience interacts with motion and attentional allocation. Future research would benefit from adopting more continuous or multi-level measures of familiarity to examine how the effects of motion vary across different stages of face learning. Finally, the control sample was disproportionately female, which may limit the generalisability of the findings.

## 5. Conclusions

In summary, the present series of experiments shows that for both typical recognisers and individuals with DP, motion facilitates familiar face recognition and unfamiliar face learning, and that this advantage is accompanied by systematic changes in visual attention. Across both experiments and participant groups, motion drew attention towards the internal facial features and reduced attentional shifting across the face, findings that are consistent with the attentional assumptions of the SSH. At the same time, the pattern of results suggests that attention alone is unlikely to provide a complete explanation for the motion advantage. Rather, the effects of motion are likely to reflect the combined contribution of attentional, representational, and identity-specific dynamic cues, with their relative importance varying across familiar and unfamiliar face processing.

## Figures and Tables

**Table 1:** Mean (SD) proportional dwell time and fixation count to each IA as a function of presentation style.

Measure	IA	Static	Moving
Dwell Time	Internal	56.38 (14.51)	61.22 (13.94)
	External	38.99 (13.35)	33.11 (12.21)
	Eyes	19.41 (14.23)	20.98 (15.66)
	Nose	26.18 (14.15)	26.88 (13.22)
	Mouth	10.79 (12.08)	13.35 (14.18)
Fixation Count	Internal	51.12 (11.96)	64.80 (14.40)
	External	42.43 (11.11)	31.07 (11.95)
	Eyes	16.60 (10.88)	20.15 (13.67)
	Nose	23.79 (10.60)	28.26 (12.72)
	Mouth	10.74 (9.82)	16.38 (11.63)

**Table 2:** Mean (SD) proportional dwell time and fixation count to each IA as a function of presentation style and group.

		DP		Control	
Measure	IA	Static	Moving	Static	Moving
Dwell Time	Internal	58.22 (12.51)	64.9 (8.04)	63.66 (9.87)	65.46 (11.76)
	External	37.87 (9.38)	31.76 (7.81)	31.28 (9.7)	30.35 (11.95)
Fixation Count	Internal	53.39 (10.8)	57.43 (8.75)	67.63 (8.83)	68.2 (7.78)
	External	41.18 (8.2)	37.66 (8.42)	28.92 (8.06)	29.6 (8.2)
	Eyes	16.57 (9.89)	7.58 (7.75)	22.19 (11.26)	10.71 (10.72)
	Nose	25.6 (8.1)	24.22 (10.96)	28.89 (8.83)	28.71 (9.93)
	Mouth	11.21 (9.85)	25.63 (16.51)	16.55 (12.8)	28.77 (16.81)

**Table 3:** Mean (SD) proportional dwell time and fixation count to each IA during the learning phase as a function of presentation style.

Measure	IA	Static	Moving
Dwell Time	Internal	76.52 (10.79)	79.55 (11.36)
	External	22.75 (10.31)	18.74 (11.1)
	Eyes	35.64 (16.82)	37.14 (17.58)
	Nose	24.02 (13.48)	23.3 (15.96)
	Mouth	16.87 (10.7)	19.12 (13.7)
Fixation Count	Internal	74.37 (11.5)	78.03 (11.3)
	External	24.86 (10.58)	21.17 (10.91)
	Eyes	31.75 (13.5)	32.71 (14.64)
	Nose	26.84 (12.76)	27.6 (13.88)
	Mouth	15.78 (9.28)	17.73 (10.44)

**Table 4:** Mean (SD) proportional dwell time and fixation count to the internal and external IAs during face recognition as a function of presentation style at learning.

Measure	IA	Static	Moving
Dwell Time	Internal	79.12 (11.66)	81.54 (11.25)
	External	20.63 (11.75)	18.08 (11.16)
Fixation Count	Internal	77.34 (11.16)	79.71 (11.97)
	External	22.23 (11.35)	19.7 (11.57)

**Table 5:** Mean (SD) proportional dwell time and fixation count to each IA during face learning as a function of presentation style and group.

		DP		Control	
Measure	IA	Static	Moving	Static	Moving
Dwell Time	Internal	62.76 (13.79)	66.91 (15.57)	80.07 (10.17)	81.43 (9.92)
	External	36.58 (13.77)	30.90 (15.33)	19.17 (9.65)	16.70 (9.39)
Fixation Count	Internal	61.58 (12.18)	66.43 (12.87)	78.23 (10.29)	80.63 (9.13)
	External	37.69 (12.20)	32.86 (12.70)	20.77 (8.95)	18.41 (8.11)
	Eyes	20.18 (12.07)	21.24 (12.73)	35.21 (11.41)	36.02 (12.43)
	Nose	22.92 (6.02)	25.75 (9.28)	27.40 (11.80)	28.04 (14.08)
	Mouth	18.47 (9.94)	19.45 (8.81)	15.62 (7.24)	16.57 (9.40)

**Table 6:** Mean (SD) proportional dwell time and fixation count to IAs (internal and external features) during face recognition as a function of presentation style and group.

		DP		Control	
Measure	IA	Static	Moving	Static	Moving
Dwell Time	Internal	69.54 (16.15)	67.92 (9.69)	80.57 (13.62)	82.57 (8.71)
	External	29.89 (15.83)	31.36 (10.05)	18.97 (13.66)	16.57 (8.83)
Fixation Count	Internal	68.54 (13.87)	65.82 (9.77)	78.08 (12.03)	81.04 (9.76)
	External	30.52 (13.50)	32.89 (10.44)	21.13 (12.22)	17.79 (9.28)

## Data Availability

The data presented in this study are available on request from the corresponding author. The data are not publicly available due to ethical restrictions as participants did not consent to the data being shared publicly.

## Abbreviations

The following abbreviations are used in this manuscript:

ANOVA	Analysis of Variance
ASD	Autism spectrum disorder
BORB	Birmingham Object Recognition Battery
CRT	Cathode Ray Tube
DP	Developmental Prosopagnosia
DV	Dependent Variable
GIMP	GNU Image Manipulation Programme
IA	Interest Area
IV	Independent Variable
REH	Representation Enhancement Hypothesis
SIH	Supplemental Information Hypothesis
SSH	Social Signals Hypothesis
